# IGF-1 boosts mitochondrial function by a Ca^2+^ uptake-dependent mechanism in cultured human and rat cardiomyocytes

**DOI:** 10.3389/fphys.2023.1106662

**Published:** 2023-02-08

**Authors:** Pablo Sánchez-Aguilera, Camila López-Crisosto, Ignacio Norambuena-Soto, Christian Penannen, Jumo Zhu, Nils Bomer, Matijn F. Hoes, Peter Van Der Meer, Mario Chiong, B. Daan Westenbrink, Sergio Lavandero

**Affiliations:** ^1^ Advanced Center for Chronic Diseases (ACCDiS), Facultad de Ciencias Químicas y Farmacéuticas, Facultad de Medicina, Universidad de Chile, Santiago, Chile; ^2^ Department of Cardiology, University Medical Center Groningen, University of Groningen, Groningen, Netherlands; ^3^ Department of Clinical Genetics, Maastricht University Medical Center, Maastricht, Netherlands; ^4^ Department of Genetics and Cell Biology, Faculty of Health, Medicine and Life Sciences, Maastricht University, Maastricht, Netherlands; ^5^ CARIM School for Cardiovascular Diseases, Maastricht, Netherlands; ^6^ Cardiology Division, Department of Internal Medicine, University of Texas Southwestern Medical Center, Dallas, TX, United States

**Keywords:** insulin-like growth factor 1 (IGF-1), MCU complex, mitochondrial calcium handling, physiological cardiac hypertrophy, neonatal rat ventricular myocytes (NRVMs), human embryonic stem cell derived-cardiomyocytes (hES-CMs)

## Abstract

A physiological increase in cardiac workload results in adaptive cardiac remodeling, characterized by increased oxidative metabolism and improvements in cardiac performance. Insulin-like growth factor-1 (IGF-1) has been identified as a critical regulator of physiological cardiac growth, but its precise role in cardiometabolic adaptations to physiological stress remains unresolved. Mitochondrial calcium (Ca^2+^) handling has been proposed to be required for sustaining key mitochondrial dehydrogenase activity and energy production during increased workload conditions, thus ensuring the adaptive cardiac response. We hypothesized that IGF-1 enhances mitochondrial energy production through a Ca^2+^-dependent mechanism to ensure adaptive cardiomyocyte growth. We found that stimulation with IGF-1 resulted in increased mitochondrial Ca^2+^ uptake in neonatal rat ventricular myocytes and human embryonic stem cell-derived cardiomyocytes, estimated by fluorescence microscopy and indirectly by a reduction in the pyruvate dehydrogenase phosphorylation. We showed that IGF-1 modulated the expression of mitochondrial Ca^2+^ uniporter (MCU) complex subunits and increased the mitochondrial membrane potential; consistent with higher MCU-mediated Ca^2+^ transport. Finally, we showed that IGF-1 improved mitochondrial respiration through a mechanism dependent on MCU-mediated Ca^2+^ transport. In conclusion, IGF-1-induced mitochondrial Ca^2+^ uptake is required to boost oxidative metabolism during cardiomyocyte adaptive growth.

## 1 Introduction

Cardiac hypertrophy is a type of cardiac remodeling characterized by the enlargement of the heart in response to a repetitive or sustained increase in cardiac workload, classified as either physiological or pathological (Hill and Olson, 2008). Physiological cardiac hypertrophy develops during exercise or pregnancy and is characterized by mild reversible cardiac growth with conserved or increased contractility (Nakamura and Sadoshima, 2018). In contrast, pathological cardiac hypertrophy is an excessive cardiac growth observed in most cardiovascular diseases due to an unremitting increase in cardiac workload, which results in progressive and irreversible contractile dysfunction, cell death, and fibrosis (Nakamura and Sadoshima, 2018). There is little agreement on the mechanisms that determine both hypertrophic phenotypes, which could explain the lack of efficient treatments for pathological remodeling that often leads to heart failure (Hill and Olson, 2008).

Both hypertrophies are related to different signaling pathways and have a wholly opposed metabolic signature and energetic balance at the cardiomyocyte level (Brown et al., 2017). There is a consensus that physiological cardiac hypertrophy requires the activation of IGF-1 receptor signaling and mitochondrial function to be adaptive (Troncoso et al., 2014; Nijholt et al., 2022). In contrast, mechanical stress and catecholamines/angiotensin II overstimulation have been associated with Ca^2+^ mishandling and mitochondrial dysfunction, which are common features of pathological cardiac hypertrophy (Crilley et al., 2003; Nakamura and Sadoshima, 2018). Mitochondrial function and the energetic status of cardiomyocytes appear to be critical factors determining the developing hypertrophic phenotype.

Ca^2+^ exerts a delicate control of the energetic balance in cardiomyocytes. It promotes ATP consumption during contraction and increases mitochondrial ATP production to meet cardiac energy demands (Glancy and Balaban, 2012). This process occurs during each action potential when the sarcoplasmic reticulum releases Ca^2+^ that is partially absorbed by the mitochondria, enhancing the pyruvate metabolism and the tricarboxylic acid (TCA) cycle activity, thus promoting ATP synthesis (Denton, 2009). To reach the mitochondrial matrix, Ca^2+^ crosses the inner mitochondrial membrane through the mitochondrial Ca^2+^ uniporter (MCU) complex, strategically positioned in the vicinity of the junctional sarcoplasmic reticulum-mitochondrial associations (De La Fuente et al., 2016). The MCU complex (MCUC) is a Ca^2+^ transporter formed by the pore-forming subunit MCU, its paralog MCUB and the essential MCU regulator (EMRE) (Fan et al., 2020). In addition, MCU-mediated Ca^2+^-transport is regulated by mitochondrial Ca^2+^ uptake 1 and 2 (MICU1/2) and the MCU regulator 1 (MCUR1), which respectively control the channel gating and the conductance of MCU (Tomar et al., 2016; Wang et al., 2020).

Recently, the MCUC was linked to physiological cardiac hypertrophy. MCU protein levels increased in parallel with cardiac growth in two models of adaptive cardiac hypertrophy (Zaglia et al., 2017). Moreover, cardiac-specific overexpression of MCU in mice with pathological hypertrophy increased mitochondrial respiration and recovered cardiac performance, thereby resembling physiological cardiac growth (Suarez et al., 2018; Liu et al., 2021). There is plenty of evidence that mitochondrial dysfunction and Ca^2+^ mishandling contribute to the pathological cardiac hypertrophy (Gorski et al., 2015; Ljubojevic-Holzer et al., 2020; Liu et al., 2021). However, little attention has been paid to mitochondrial Ca^2+^ handling and its influence on oxidative metabolism in models of physiological cardiac hypertrophy. To address this question, we studied the effect of the physiological hypertrophic inductor IGF-1 on mitochondrial Ca^2+^ handling and oxidative metabolism in cultured cardiomyocytes.

## 2 Materials and methods

### 2.1 Neonatal rat ventricular myocytes (NRVMs) culture

NRVMs were obtained from 2 to 3 days old Sprague-Dawley rats. The isolation and culture were developed according to Galvez et al. (Galvez et al., 2001). Briefly, neonatal rats were decapitated, hearts extracted, and atria removed. Ventricles were washed in sterile Hank’s medium (Sigma-Aldrich, San Luis, MI, United States) at 37°C and submitted to enzymatic digestion. Cardiomyocytes were purified by a pre-pleated step for 2 h at 37°C to remove fibroblasts and afterward cultured in maintenance medium (DME:M199, 4:1; Sigma-Aldrich) supplemented with 15% fetal bovine serum and 0.1 mM bromodeoxyuridine (Sigma-Aldrich).

### 2.2 Human embryonic stem cells derived cardiomyocytes (hES-CMs) culture

HUES9 human embryonic stem cells from Harvard Stem cells institute were differentiated into cardiomyocytes using a small molecule-derived approach (Hoes et al., 2018; Bomer et al., 2020). Briefly, HUES9 cells were maintained in Essential 8 medium (A1517001; Thermo Fisher Scientific, Waltham, MA, United States) before differentiation to cardiomyocytes was initiated, which was achieved by culturing HUES9 cells in RPMI1640 medium (21875-034, Life Technologies, Carlsbad, CA, United States) supplemented with 1x B27 minus insulin (Life Technologies, Carlsbad, CA, United States) and 6 μmol/L CHIR99021 (13122, Cayman Chemical, Ann Arbor, MI, United States). After 2 days, medium was refreshed with RPMI1640 supplemented with 1x B27 minus insulin and 2 μmol/L Wnt-C59 (5148, Tocris Bioscience, Bristol, United Kingdom). After 2 days, medium was changed to CDM3 medium (RPMI1640 plus 213 μg/mL ascorbic acid 2-phosphate plus 500 μg/mL recombinant human albumin). On day 8 after induction of differentiation, spontaneously contracting cardiomyocytes were observed, which were subsequently purified by replacing the medium to glucose-free CDM3 medium supplemented with 5 mmol/L sodium DL-lactate (CDM3L; Sigma-Aldrich). Ultimately, this resulted in >99% pure spontaneously beating cardiomyocyte cultures. The hES-CMs were suitable for experimentation ∼30 days after the differentiation protocol started.

### 2.3 IGF-1 stimulation

Cardiomyocytes were stimulated with 10 nM recombinant human IGF-1 + 0.1% BSA (Thermo Fisher Scientific, Waltham, MA, United States) or 0.1% BSA in CDM3 or maintenance medium (hES-CMs or NRVMs respectively) between 15 min and 48 h depending on the experimental setup.

### 2.4 Lentiviral production and transduction of hES-CMs

HEK-293T cells were cultured at 37°C, and 5% CO_2_ in Dulbecco modified Eagle medium (DMEM; Thermo Fisher Scientific) supplemented with 10% fetal calf serum (Sigma-Aldrich). At 70% confluence, the cells were transfected with Fugene HD (Promega, Madison, WI, United States) together with pCMV∆8.91-transfer plasmid, VSV-G-packaging plasmid, and pLKO.1-plasmid expressing the genetically encoded mitochondrial Ca^2+^ indicator Mitycam provided by Dr. Adam Cohen (pMOS028, Addgene, plasmid #163046). The medium was replaced with CDM3 after 24 h and the supernatant containing the viral particles was harvested and filtered with a 0.45 nm filter after 48 h hES-CMs were incubated with CDM3 supplemented with 40% clean viral supernatant for 24 h. The next day, the medium was replaced by CMD3. After 48 h, cells were available for experiments.

### 2.5 Mitochondrial Ca^2+^ uptake

#### 2.5.1 hES-CMs

Transfected hES-CMs with the Ca^2+^ indicator Mitycam were incubated in Tyrode medium (130 mM NaCl, 4 mM KCl, 2 mM CaCl_2_, 1 mM MgCl_2_, 10 mM glucose, 10 mM HEPES, pH 7.2) and mounted at 37°C in the Olympus IX-71 inverted microscope DeltaVision Elite (Olympus, Tokyo, Japan). Mitycam fluorescence (excitation/emission spectra: 498/515) was recorded at baseline (5 min) and during electric stimulation (2 Hz, 6 min) in the presence of 10 nM isoproterenol (Sigma-Aldrich).

#### 2.5.2 NRVMs

Mitochondrial Ca^2+^ uptake was determined using the Ca^2+^ indicator Rhod2-AM (excitation/emission spectra: 575/675. Invitrogen, Waltham, MA, United States) (Maxwell et al., 2018). Briefly, cells were incubated with Rhod2-AM in Tyrode medium for 30 min at room temperature, washed, and de-esterified for another 30 min. Subsequently, NRVMs were permeabilized with 0.005% saponin for 30 s followed by the replacement for an internal medium without Ca^2+^. Cells were mounted in the Zeiss LSM 700 laser scanning confocal microscope (Carl Zeiss AG, Oberkochen, Germany), and the baseline Rhod2 fluorescence was recorded for 20 s. The medium was replaced by an internal medium with 2 µM free Ca^2+^, and the signal was recorded for 100 s.

For both cell types (hES-CMs and NRVMs), the images were processed and analyzed in the open-source software Fiji (Schindelin et al., 2012). For imaging analysis, the background signal was subtracted. Mitycam fluorescence signal was expressed as 1—(F/F0) and Rhod2 fluorescence signal as F/F0.

### 2.6 Cell lysate and protein extraction

Cardiomyocytes were lysed in RIPA buffer supplemented with protease inhibitors (Roche, Basile, Switzerland), phosphatase inhibitors (Sigma-Aldrich), sodium orthovanadate (Sigma-Aldrich), and phenylmethylsulphonyl fluoride (Roche). Subsequently, cells were centrifuged at 12,000 g for 10 min at 4°C. The supernatant was recovered, and the isolated protein was quantified using the BCA protein assay (Thermo Fisher Scientific). The protein extract was cooked at 95°C for 5 min in SDS-PAGE loading buffer (Thermo Fisher Scientific) and stored at −20°C.

### 2.7 Electrophoresis, electro-transference, and western blotting

Protein electrophoresis was carried out by standard methods. Briefly, SDS PAGE was loaded with 10 µg of protein extract. Proteins were separated by electrophoresis at 80 mV in running buffer and electro-transferred to a PVDF membrane at 0.45 A for 90 min in transference buffer. Primary antibodies were diluted in blocking buffer (TBS, 0.1% Tween, and 5% bovine serum albumin) in a 1:1,000 ratio and incubated overnight at 4°C. Membranes were washed in TBS-0.1% tween and incubated in blocking buffer with the secondary antibody (anti-mouse or anti-rabbit, Sigma-Aldrich) for 1 h in a 1:5,000 ratio. REVERT™ Total protein staining was used as loading control (LI-COR Lincoln, NE, United States). Western blot detection was carried out in a chemiluminescence detector by standard methods.

### 2.8 Reverse transcription and real-time qPCR

Cells were lysed with TRIzol^®^ (Invitrogen, Carlsbad, CA, United States) following the manufacturer’s instructions. The mRNA was quantified using a NanoDrop 2000 machine (Thermo Fisher Scientific). The cDNA was synthesized with the Quantitect RT kit (Qiagen N.V., Hilden, Germany) following the manufacturer’s instructions. All primers were designed in Primer-Blast software (NCBI, Bethesda, MD, United States) and internally validated. qPCR was carried out using the SYBR^®^ Green Master Mix (Bio-Rad, Hercules, CA, United States) in the thermocycler CFX384 Touch Real-Time PCR Detection System (Bio-Rad).

### 2.9 Determination of mitochondrial membrane potential

Cardiomyocytes were incubated with 20 nM tetramethylrhodamine ethyl ester (TMRE^+^, Invitrogen; excitation/emission spectra: 553/577) in Tyrode medium at 37°C for 30 min. Cardiomyocytes were mounted in the Olympus IX-71 inverted microscope DeltaVision Elite. Baseline fluorescence was recorded for ∼200 s and subsequently, exposed to 10 mM Carbonyl cyanide-p-trifluoromethoxyphenylhydrazone (FCCP) for ∼1,000 s. The noise signal was subtracted. TMRE^+^ fluorescence was expressed as F—F_min_ (relative fluorescence unit, RFU).

### 2.10 Mitochondrial respiration assay (seahorse)

hES-CMs were seeded in Seahorse assay plates at a density of 0.25 x 10^5^ cells/well. 24 h before the experiment, cardiomyocytes were stimulated with IGF-1 or IGF-1 + 10 µM ru360 (Merck, Darmstadt, Germany). Oxygen consumption rate was determined by the Seahorse XF Cell Mito Stress Test programmed in the extracellular metabolic flux analyzer Agilent Seahorse XF96 (Agilent Technologies, Santa Clara, CA, United States). 20 min before the assay, CDM3 medium was replaced by Seahorse assay medium XF (Agilent Technologies) supplemented with 10 mM glucose and 1 mM sodium pyruvate at 37°C without CO_2_ control. The standard stress protocol was performed (Hoes et al., 2018), and the oxygen consumption rate was normalized by protein content determined by BCA protein assay.

### 2.11 Cell viability assay

Cell viability was determined using the Real-Time-Glo™ MT Cell viability assay Kit (Promega, Madison, WI, United States) following the manufacturer’s instructions. Briefly, the NanoLuc^®^ enzyme and the viability substrate were 1x diluted and equilibrated at 37°C in CDM3 medium or CDM3 medium +10 µM ru360 (experimental media). hES-CMs were seeded in a clear bottom 96 well plate. CDM3 medium was replaced by the corresponding experimental medium and the luminescence was recorded from 0 to 48 h in the Synergy H1 plate reader (BioTek, Winooski, VT, United States). The signal was corrected by the protein content in each well.

### 2.12 Cell size determination

NRVMs were seeded in gelatin-coated coverslips in a maintenance medium and stimulated with IGF-1 for 48 h. Subsequently, NRVMs were fixed in PBS + 4% paraformaldehyde for 15 min. Afterward, cells were incubated with rhodamine-phalloidin (1:500, Thermo Fisher Scientific) and Hoescht (1:1,000, Thermo Fisher Scientific) for 1 h at room temperature. Coverslips were mounted in slides with DAKO mounting medium (Agilent Technologies). The images were acquired using the Zeiss LSM 700 laser scanning confocal microscope. The relative cell area was quantified using the software Fiji. 50 to 100 cells were analyzed per condition.

### 2.13 Statistical analysis

All the results were expressed as mean ± SE from at least three independent assays. For values with normal distribution and equal variances was used *t*-test or one-way ANOVA was followed by the Tukey post-hoc test for multiple comparisons. For comparing groups without normal distribution was used U Mann-Whitney test or the Kruskal–Wallis test followed by the Dunn post-hoc test for multiple comparisons. A *p*-value < 0.05 was considered for statistical differences. The software GraphPad Prism 9 (GraphPad Software Inc., San Diego, CA, United States) was used for data analysis and visualization.

## 3 Results

### 3.1 IGF-1 increases mitochondrial Ca^2+^ uptake and PDH activity in human and rat cardiomyocytes

**FIGURE 1:**
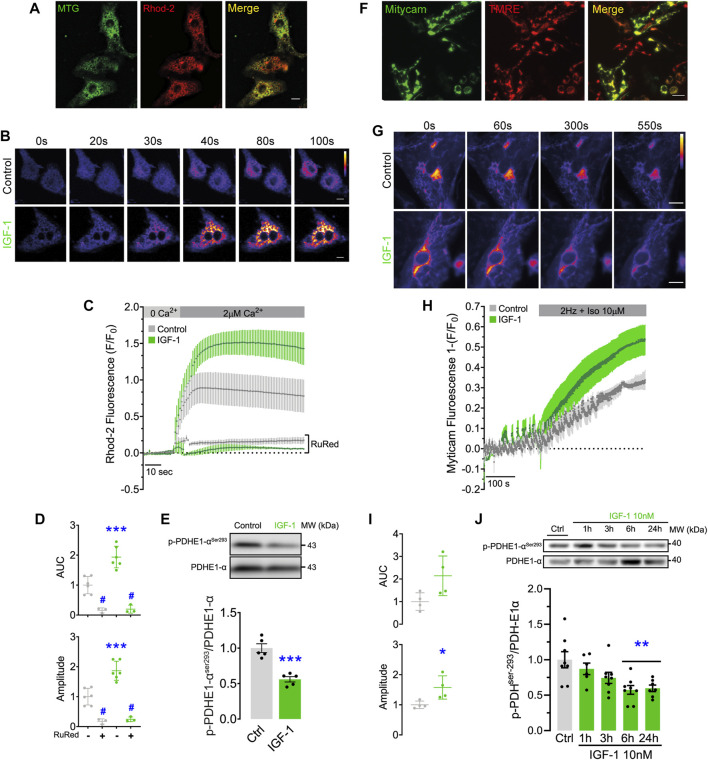
IGF-1 increases mitochondrial Ca^2+^ uptake and reduces the PDH phosphorylation in NRVMs and hES-CMs. **(A)** Rhod2 and Mitotracker Green fluorescence pattern. Scale bar: 20 µm. **(B)** Representative confocal images of Rhod2 fluorescence were recorded for 100 s in permeabilized NRVMs treated with IGF-1 for 24 h and exposed to a pulse of 2 μM free Ca^2+^ (*n* = 3–6). Scale bar: 20 µm. **(C)** Rhod2 fluorescence kinetic obtained from B, including ruthenium red (RuRed, 5 μM) treated conditions. **(D)** Area under the curve and maximal fluorescence (amplitude) registered in C for each condition. **(E)** Representative Western blot and quantification of phosphorylated PDH in serine 293 in NRVMs treated with IGF-1 for 24 h (*n* = 6). *p < 0.05 vs. control **p < 0.01 vs. control. ***p < 0.001 vs. control; #*p* < 0.05 vs. non-RuRed treated condition. **(F)** TMRE^+^ and Mitycam fluorescence pattern. Scale bar: 10 µm. **(G)** Representative images of Mitycam fluorescence were recorded for 550 s in hES-CMs treated with IGF-1 for 24 h and exposed to an electric field stimulation (2 Hz) and isoproterenol (Iso, 10 µM). (*n* = 3—6). Scale bar: 10 µm. **(H)** Mitycam fluorescence kinetic. **(I)** Area under the curve and maximum fluorescence (amplitude) determined from H for each condition (*n* = 4). **(J)** Representative Western blot and quantification of phosphorylated PDH in serine 293 between 1 and 24 h of IGF-1 stimulation (*n* = 7—8).

To study mitochondrial Ca^2+^ handling under conditions of physiological hypertrophy, we studied Ca^2+^ uptake kinetics of NRVMs in response to IGF-1 stimulation. We first corroborated the activation of IGF-1 receptor-dependent signaling in these cells and observed a time-dependent increase in AKT phosphorylation (Supplementary Figure S1). IGF-1 also promoted a clear hypertrophic response in NRVMs (Supplementary Figure S2). To assess the effect of IGF-1 on mitochondrial Ca^2+^ uptake we made use of the Ca^2+^ indicator Rhod2, restricting the fluorescence signal into the mitochondria by a permeabilization step with saponin (Figure 1A). IGF-1-treated NRVMs significantly improved their Ca^2+^ uptake capacity after an extracellular pulse of 2 µM free Ca^2+^ (Figures 1B, C), observed as higher maximal fluorescence and area under the curve than the control condition (Figure 1D). As a control, we blocked mitochondrial Ca^2+^ uptake with the MCU inhibitor ruthenium red (RuRed), confirming the role of the MCUC on the observed effect (Figures 1C, D). The rise in mitochondrial Ca^2+^ concentration activates the pyruvate dehydrogenase (PDH) phosphatase, which dephosphorylates and activates the PDH complex (Denton, 2009). We found that IGF-1 stimulation significantly reduced the PDH phosphorylation by 0.55 ± 0.04-fold over the control condition (Figure 1E).

To generalize the results found in NRVMs, we replicated our findings in hES-CMs. Similar to NRVMs, IGF-1 induced AKT phosphorylation in these cells (Supplementary Figure S1). To measure the mitochondrial Ca^2+^ uptake, hES-CMs were infected with a lentiviral vector containing the genetically encoded Ca^2+^ indicator Mitycam, whose expression is restricted to mitochondria. As shown in Figure 1F, the Mitycam signal has an almost identical fluorescence pattern to TMRE^+^, a potentiometric dye that accumulates within the mitochondrial matrix. We promoted Ca^2+^ release from the sarcoplasmic reticulum by using electric field stimulation in presence of β-adrenergic agonist isoproterenol (Iso). We observed a drop in Mitycam fluorescence during electric stimulation, which was more pronounced at the end of the protocol in the cells treated with IGF-1 (Figures 1G–I), suggesting a greater capacity for Ca^2+^ uptake. As well as in NRVMs, IGF-1 stimulation significantly reduced the PDH phosphorylation (∼43% y ∼41% at 6 and 24 h of incubation, respectively) compared to non-treated cells (Figure 1J), indicative of a higher PDH activity.

Overall, these data suggest that IGF-1 enhances mitochondrial Ca^2+^ handling and pyruvate metabolism in both cellular models, indicating a conserved response in both species.

### 3.2 IGF-1 modifies the expression of MCUC elements and increases the mitochondrial membrane potential

**FIGURE 2:**
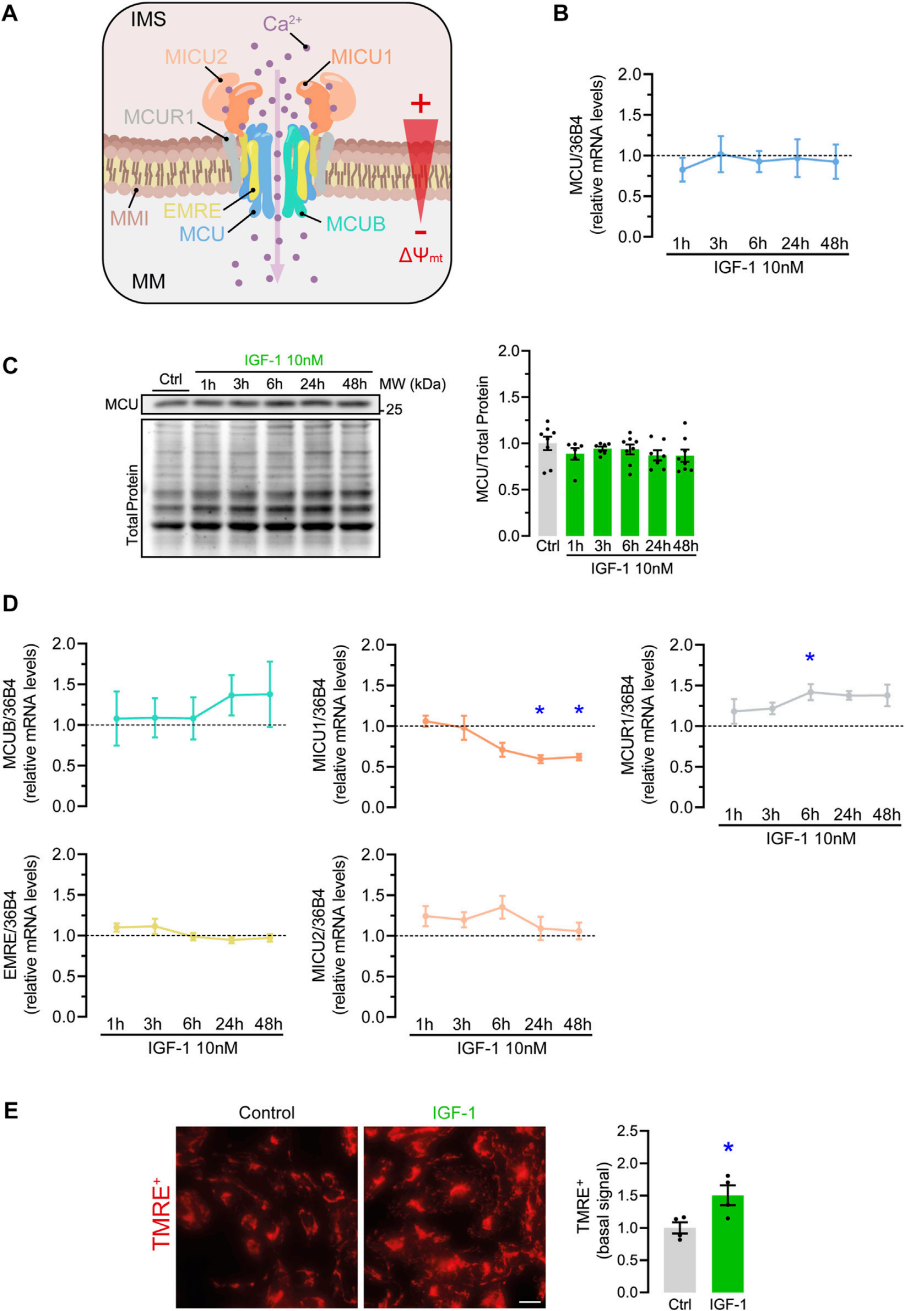
Effect of IGF-1 on MCU complex elements expression and ∆Ψ_mt_ in hES-CMs. **(A)** Scheme of MCU complex components (MCU, MCUB, SMDT1, MICU1, MICU2, MCUR1) inserted in the inner mitochondrial membrane (IMM) and a representation of the ∆Ψ_mt_ between the intermembrane space (IMS) and the mitochondrial matrix (MM); **(B)** Relative mRNA and **(C)** protein levels of MCU in hES-CMs stimulated with IGF-1 for 1–48 h (*n* = 6—8); **(D)** Relative mRNA levels of MCU complex regulatory subunits (MCUB, SMDT1, MICU1, MICU2, MCUR1) in hES-CMs stimulated with IGF-1 for 1–48 h (*n* = 6—8). The dotted line represents the mRNA levels of the untreated (control) condition; **(E)** Relative levels of basal TMRE^+^ in cells treated with IGF-1 for 24 h *p < 0.05 vs. control. Scale bar: 10 µm.

The limiting pathway of mitochondrial Ca^2+^ influx is the MCUC. Its function is mainly regulated by changes in the expression of the MCUC subunits affecting its Ca^2+^ sensitivity, gating, or the stability of the complex (Figure 2A) (Feno et al., 2021). In hES-CMs, IGF-1 did not change either MCU mRNA (Figure 2B) or protein levels (Figure 2C) at any of the evaluated time points. However, compatible with a higher MCU conductance, we observed a significant reduction of MICU1 mRNA levels at 24 and 48 h (0.59 ± 0.05 and 0.61 ± 0.04-fold over control conditions, respectively) and increased mRNA levels of MCUR1 at 6 h (1.41 ± 0.1-fold over control condition) (Figure 2D).

Next to MCUC composition, Ca^2+^ transport also depends on an electric gradient between the inter-membrane space and the mitochondrial matrix, known as mitochondrial membrane potential (∆Ψ_mt_). This is the main driving force for Ca^2+^ influx through the MCUC (Figure 2A) (Nicholls and Crompton, 1980). Therefore, an increment of ∆Ψ_mt_ will promote a higher MCU-mediated Ca^2+^ transport. To test this hypothesis, we determined the basal ∆Ψ_mt_ in TMRE^+^-loaded cardiomyocytes and found that, indeed, IGF-1 increased the basal ∆Ψ_mt_ by 1.5 ± 0.09-fold over the control condition (Figure 2E). Altogether, these results indicate that IGF-1 enhances mitochondrial Ca^2+^ handling by a mechanism that combines changes in the MCUC and the driving force for mitochondrial Ca^2+^ influx.

### 3.3 IGF-1 increases cardiomyocyte respiration by a mechanism dependent on mitochondrial Ca^2+^ uptake

**FIGURE 3:**
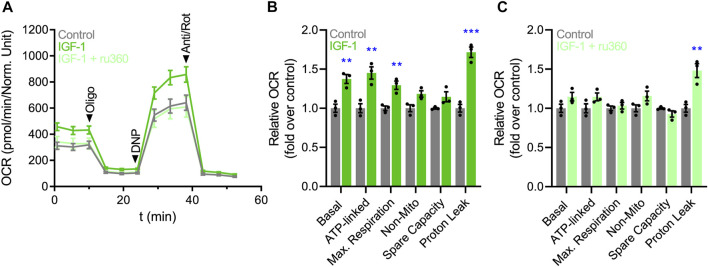
IGF-1 increases mitochondrial respiration by a mechanism dependent on mitochondrial Ca^2+^ uptake. **(A)** Representative plot of OCR over time. **(B)** Quantification of the relative changes in OCR (basal respiration, ATP-linked respiration, maximal respiration, non-mitochondrial respiration, spare capacity, and proton leak) in hES-CMs stimulated with IGF-1 (10 nM for 24 h) or vehicle. **(C)** Quantification of the relative changes in OCR in hES-CMs stimulated with ru360 (10 µM) or vehicle respiration (*n* = 3).

The elevation of mitochondrial Ca^2+^ concentrations promotes the activity of PDH and the TCA cycle, indirectly increasing oxygen consumption rate (OCR) and ATP synthesis (Williams et al., 2015). Based on this premise, we investigated the effect of IGF-1 on the OCR of hES-CMs, using an extracellular metabolic flux analyzer. As expected, IGF-1 increased basal (1.37 ± 0.10-fold), ATP-linked (1.45 ± 0.14-fold), and maximal respiration (1.29 ± 0.09-fold), including the proton leak (1.71 ± 0.12-fold) (Figures 3A, B). To confirm whether the mitochondrial Ca^2+^ could play a role in the IGF-1-mediated mitochondrial respiration, we evaluated the OCR in hES-CMs co-incubated with the specific MCU inhibitor ru360. Remarkably, ru360 completely prevented the changes in OCR induced by IGF-1, showing similar values as the control condition (Figures 3A–C), except for the proton leak, which remained high (1.48 ± 0.15-fold over the control condition). It is important to note that in all evaluated time points, ru360 did not reduce cell viability (Supplementary Figure S3). Altogether, these results suggest that IGF-1 increases mitochondrial respiration by a mechanism dependent on MCU-mediated Ca^2+^ uptake.

## 4 Discussion

Our study showed that IGF-1 boosts mitochondrial Ca^2+^ uptake and stimulates Ca^2+^-sensitive mitochondrial dehydrogenases, which enhances oxidative metabolism during cardiomyocyte growth in both NRVMs and hES-CMs. Moreover, we propose that IGF-1 regulates mitochondrial Ca^2+^ uptake through modifications in MCUC composition and increasing ∆Ψ_mt_. This study suggest that physiological cardiomyocyte growth is strictly linked to mitochondrial function and energy homeostasis and highlight Ca^2+^ as the main signal to allow the energetic adaptation.

Ca^2+^ allows the real-time adaptation of mitochondrial function to meet energy demands during increased cardiomyocyte workload (Wescott et al., 2019). This occurs even under pathological cardiac remodeling where MCU upregulation sustains mitochondrial and cardiac function and its downregulation exacerbates the pathological phenotype (Wang et al., 2022). This is possible because part of the released Ca^2+^ during contraction is absorbed by mitochondria, directly stimulating the activity of the isocitrate and α-ketoglutarate dehydrogenases, and indirectly, the PDH activity, which together will increase ATP synthesis (Glancy and Balaban, 2012). We postulated that IGF-1 promotes mitochondrial Ca^2+^ uptake to sustain the energetic requirements of a growing cardiomyocyte. In line with this hypothesis, we observed that the activation of the IGF-1 signaling pathway increased mitochondrial Ca^2+^ uptake in both cell types (Figures 1C, H). Moreover, IGF-1 reduced phospho-PDH, which indicates higher enzymatic activity and pyruvate oxidation and, indirectly, higher mitochondrial Ca^2+^ concentration (Figures 1E,J).

Our findings add new evidence on the subacute effect of IGF-1 on mitochondrial Ca^2+^ handling, a controversial area of research. For instance, Gutiérrez et al. concluded that the stimulation with 100 nM IGF-1 do not affect mitochondrial Ca^2+^ uptake in NRVMs (Gutiérrez et al., 2014). However, this could be explained because of the usage of supraphysiological IGF-1 concentrations or the utilization of a Ca^2+^ indicator with a substantial noise signal. By contrast, Troncoso et al. showed that 10 nM IGF-1 enhanced mitochondrial function in a Ca^2+^-dependent manner during nutritional starvation in NRVMs, which is in line with our findings (Troncoso et al., 2012).

Concerning PDH, its phosphorylation status has often been used as an accepted readout of mitochondrial Ca^2+^ content, being employed in several studies to check the effect of the genetic manipulation of MCU components on mitochondrial Ca^2+^ content (Pan et al., 2013; Luongo et al., 2015; Kwong et al., 2018). Here, we show that IGF-1 regulates PDH activity, possibly by a Ca^2+^-dependent mechanism. However, this data should be taken with caution because PDH is also regulated by the pyruvate dehydrogenase kinase that is insensitive to Ca^2+^ (Matsuhashi et al., 2015).

Mitochondrial Ca^2+^ uptake is a highly regulated process that is governed by factors related to the MCUC and the electrochemical gradient for Ca^2+^ transport (Williams et al., 2013). A change in any of the MCUC components could affect the conductance or stability of the complex (Vais et al., 2015; Payne et al., 2017; Lambert et al., 2019; Van Keuren et al., 2020). In addition, the ∆Ψ_mt_ mainly generated by the electron transport chain represents the main driving force for MCU-mediated Ca^2+^ transport (Nicholls and Crompton, 1980). Interestingly, we found that IGF-1 modified both factors, the MCUC components expression and ∆Ψ_mt_ (Figure 2).

There are several combinations of the MCUC composition that are compatible with enhanced MCU activity, which could explain the higher mitochondrial Ca^2+^ uptake. As illustrated in other studies, MICU1 loss-of-function increased the basal mitochondrial Ca^2+^ content (Rao et al., 2020; Kohlschmidt et al., 2021; Singh et al., 2022) and PDH activity (Rao et al., 2020). This could be explained by the fact that MICU1 acts as the gatekeeper of the MCU channel, limiting Ca^2+^ uptake at low cytosolic Ca^2+^ concentrations (<∼1, 3 μM) (Payne et al., 2017). Therefore, a reduction in MICU1 could lead to higher resting mitochondrial Ca^2+^ concentrations, increasing PDH activity. Regarding MICUR1 function, several reports indicated that the inclusion of MCUR1 in the MCUC enhances the Ca^2+^ transport, although there is no consensus on the mechanism (Mallilankaraman et al., 2012; Tomar et al., 2016). Paupe et al. proposed that MCUR1 acted as a scaffold factor for cytochrome C oxidase, which indirectly promotes mitochondrial Ca^2+^ transport by increasing the ∆Ψ_mt_ (Paupe et al., 2015). Interestingly, this theory agrees with the higher ∆Ψ_mt_ found in our experiments.

Wescott et al. showed that mitochondrial Ca^2+^ increments are necessary and sufficient for maintaining the ∆Ψ_mt_ and ATP levels of adult cardiomyocytes during situations of high energetic demand (Wescott et al., 2019). Based on this mechanism, we proposed that mitochondrial Ca^2+^ influx promoted by IGF-1 will potentiate metabolism and respiration to sustain the energy demands imposed by the hypertrophic stimulus. As hypothesized, we found that IGF-1 significantly enhanced ATP-linked respiration in a Ca^2+^-dependent manner (Figure 3). Other studies also found that IGF-1 increases OCR in NRVMs (Troncoso et al., 2012; del Campo et al., 2014), especially when mitochondria are stimulated with pyruvate/malate (Tigchelaar et al., 2016). Interestingly, the hormone triiodothyronine, another well-described physiological hypertrophic inductor, also increases mitochondrial Ca^2+^ uptake, OCR, and ATP synthesis (Tawfik et al., 2022). In addition, we also found that IGF-1 promoted a significant increase in the proton leak relative to control cells, which did not respond to ru360 incubation. During conditions of high energy demand, the elevation of proton leak is a protective response to reduce the production of excessive reactive oxygen species (Cadenas, 2018), which could be the case of cells under hypertrophic stimulation. However, we should take these values with caution because absolute proton leak values were low, and they did not represent a significant percentage of the maximal respiration for each condition (control: 14.7 ± 3.74%; IGF-1: 13.6 ± 3.44%; IGF-1 + ru360: 16.3 ± 4.83%).

In summary, this study shows that IGF-1 boosts mitochondrial Ca^2+^ handling and improves the oxidative metabolism of cardiomyocytes during physiological growth. In addition, we identified changes in the MCUC components and ∆Ψ_mt_ as possible regulatory mechanisms of the IGF-1-mediated mitochondrial Ca^2+^ uptake. These findings shed new light on the regulatory factors of cardiomyocyte remodeling, highlighting mitochondrial Ca^2+^ handling and MCUC as potential therapeutic targets for the prevention and treatment of pathologies related to energetic imbalance, such as pathological cardiac hypertrophy and heart failure.

The major limitation of this study is the absence of a causal relationship between the changes observed in MCUC stoichiometry and the increments in mitochondrial Ca^2+^ uptake promoted by IGF-1. Further studies should focus on determining the role of MICU1 and MCUR1 on IGF-1-mediated mitochondrial Ca^2+^ uptake, using genetic approaches of gain and loss of function of these proteins. An additional limitation is the absence of direct estimations of mitochondrial ATP production, which could demonstrate that IGF-1 is responsible to maintain the energy balance during cardiomyocyte hypertrophy. Despite these limitations, our study provides deeper insight into the regulation of hypertrophy in two different cardiomyocyte models and highlights the role of mitochondrial function in the process.

## Data Availability

The raw data supporting the conclusion of this article will be made available by the authors, without undue reservation.
