# Supplementary material for: IGF-1 boosts mitochondrial function by a Ca2+ uptake-dependent mechanism in cultured human and rat cardiomyocytes
Source: Front Physiol. 2023 Feb 8;14:1106662. doi: 10.3389/fphys.2023.1106662 (PMC9944404; doi:10.3389/fphys.2023.1106662)
Supplement: Supplementary file 1 [file DataSheet1.docx]

**Supplementary Figure S1**


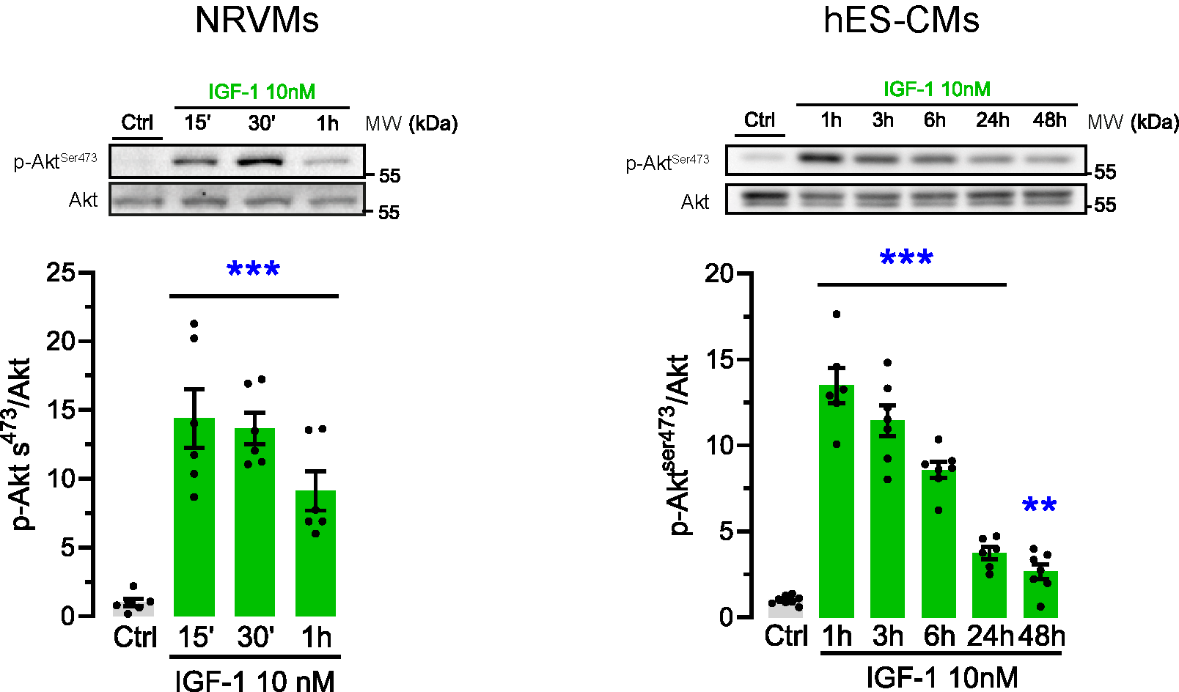


**IGF-1 stimulates AKT phosphorylation in NRVMs and hES-CMs**. Relative protein levels of phospho-AKTSer^473^ in NRVMs (n = 6) and hES-CDMs (n = 6 - 8) stimulated with 10 nM IGF-1. ** p < 0,01 vs control. *** p < 0,001 vs control.

**Supplementary Figure S2**


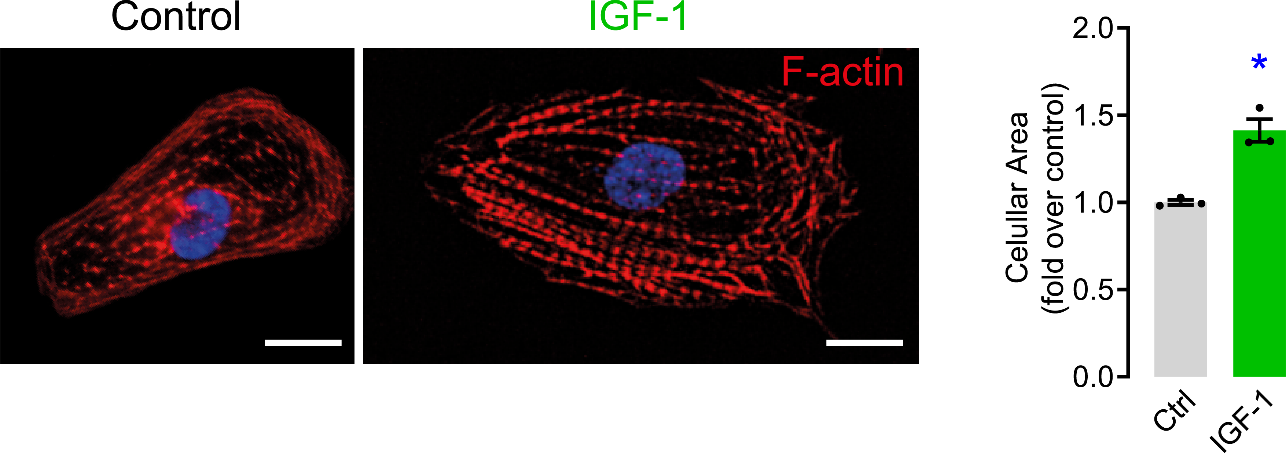


**IGF-1 promotes hypertrophy in NRVMs.** Representative confocal image showing F-actin distribution (in red) and nucleus (in blue) of NRVMs stimulated with 10 nM of IGF-1 for 24 h and quantification of cellular area (n = 3). * p < 0,05 vs control. Calibration bar: 10 µm.

**Supplementary Figure S3**


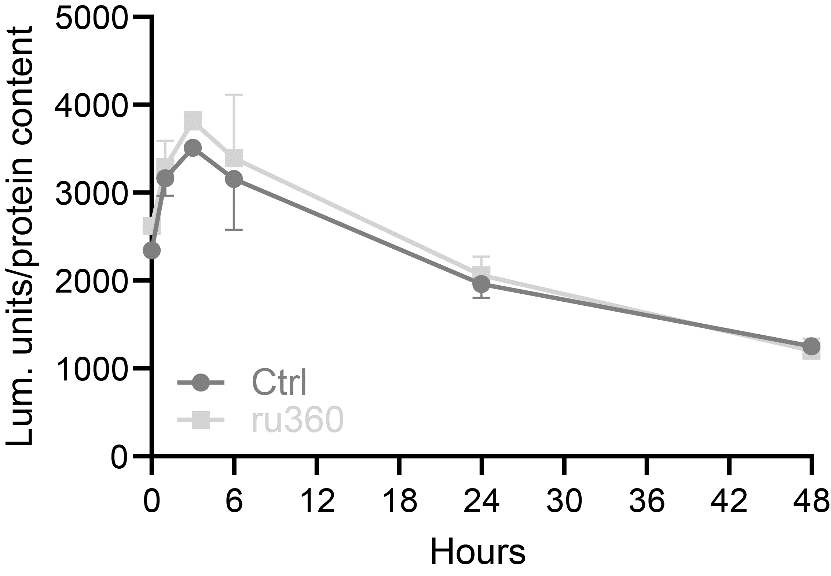


**Effect of ru360 on hES-CMs viability.** Emitted luminescence by NanoLuc® enzyme over time in hES-CMs exposed to 10 µM ru360 between 0 to 48 h (n = 3).

**Primer list**

| **Gene name** | **Forward Primer** | **Reverse Primer** |
| --- | --- | --- |
| MCU | CACTGTTGTGCCCTCTGATG | AGGCTTGAGTGTGAACTGAC |
| SDMT1 | CACTGTTGTGCCCTCTGATG | AGGCTTGAGTGTGAACTGAC |
| MICU1 | CTCAAGTCTGGATTGTGTTC | GGCGTTCAAACTCAAGCTTC |
| MICU2 | TATGACACCACGAGACTTCC | GGATCCCTGACAGTGTATCC |
| MCUB | ATAGACATGGCCTTCCCTTG | CTCAGCAGTGTGCTCATTAC |
| MCUR1 | CTGGGAGCAGGAAACTCTAC | CTCCAGGATCTTGACCAATG |
| 36B4 | AACGGGTACAAACGAGTC | AGATGGATCAGCCAAGAAG |

**Antibody list**

| **Antibody name** | **Catalog number** | **Host** |
| --- | --- | --- |
| Anti-MCU (D2Z3B) | 14997S (Cell Signalling technology) | Rabbit Monoclonal |
| anti-PDHA1 (9H9AF5) | ab110330 (Abcam) | Mouse Monoclonal |
| anti-p-PDHE1a Ser293 | NB110-93479 (Novus Biologicals) | Rabbit Polyclonal |
| anti-Akt (pan) (C67E7) | 4691 (Cell Signalling technology) | Rabbit Monocolonal |
| anti-phospho-Akt Ser473 (D9E) | 4060 (Cell Signalling technology) | Rabbit Monocolonal |
